# The long non-coding RNA H19 promotes cardiomyocyte apoptosis in dilated cardiomyopathy

**DOI:** 10.18632/oncotarget.15544

**Published:** 2017-02-20

**Authors:** Yanlin Zhang, Mengyao Zhang, Weiting Xu, Jianchang Chen, Xiang Zhou

**Affiliations:** ^1^ Department of Neurology, The Second Affiliated Hospital of Soochow University, Suzhou, China; ^2^ Department of Cardiology, The First People's Hospital of Kunshan Affiliated to Jiangsu University, Kunshan, China; ^3^ Department of Cardiology, The Second Affiliated Hospital of Soochow University, Suzhou, China

**Keywords:** H19, miR-675, PA2G4, apoptosis, dilated cardiomyopathy

## Abstract

In the previous study, we generated a rat model of dilated cardiomyopathy (DCM) induced by adriamycin and found that the expression of lncRNA H19 was significantly upregulated in myocardial tissue. The present study was aimed to investigate the potential role of H19 in the pathogenesis of adriamycin-induced DCM. H19 knockdown in the myocardium of DCM rats attenuated cardiomyocyte apoptosis and improved left ventricular structure and function. Adriamycin treatment was associated with elevated H19 and miR-675 expression and increased apoptosis in neonatal cardiomyocytes. Enforced expression of miR-675 was found to induce apoptosis in cardiomyocytes with adriamycin treatment and H19-siRNA transfection. The 3′-untranslated region of PA2G4 was cloned downstream of a luciferase reporter construct and cotransfected into HEK293 cells with miR-675 mimic. The results of luciferase assay showed that PA2G4 was a direct target of miR-675. The expression of PA2G4 was reduced in cardiomyocytes transfected with miR-675 mimic. Moreover, H19 knockdown was found to increase PA2G4 expression and suppress apoptosis in cardiomyocytes exposed to adriamycin. In conclusion, our study suggests that H19/miR-675 axis is involved in the promotion of cardiomyocyte apoptosis by targeting PA2G4, which may provide a new therapeutic strategy for the treatment of adriamycin-induced DCM.

## INTRODUCTION

Dilated cardiomyopathy (DCM) is characterized by ventricular enlargement and systolic dysfunction with reduced ventricular wall thickness [1]. It is a common cause of sudden cardiac death and congestive heart failure. A number of risk factors have been identified to be involved in the development of DCM, such as long-term use of adriamycin, one of the anthracyclines widely used for cancer chemotherapy. Adriamycin has been found to increase oxidative stress, reduce ATP production, alter gene expression, and induce mitochondrial abnormality as well as cardiomyocyte apoptosis, which are critically involved in the pathogenesis of DCM [2].

Long non-coding RNAs (lncRNAs) are transcribed RNA molecules longer than 200 nucleotides without protein-coding function. They can regulate gene expression at the epigenetic, transcriptional and post-transcriptional levels and can actively participate in various physiological and pathological processes [3]. In the previous study, we generated a rat model of DCM induced by adriamycin and found that the expression of lncRNA H19 was significantly upregulated in myocardial tissue. H19 is a highly conserved imprinted gene that plays important roles in embryonic development and growth regulation [4]. The present study was designed to investigate the pathogenic role of H19 in adriamycin-induced DCM.

## RESULTS

**Figure 1:**
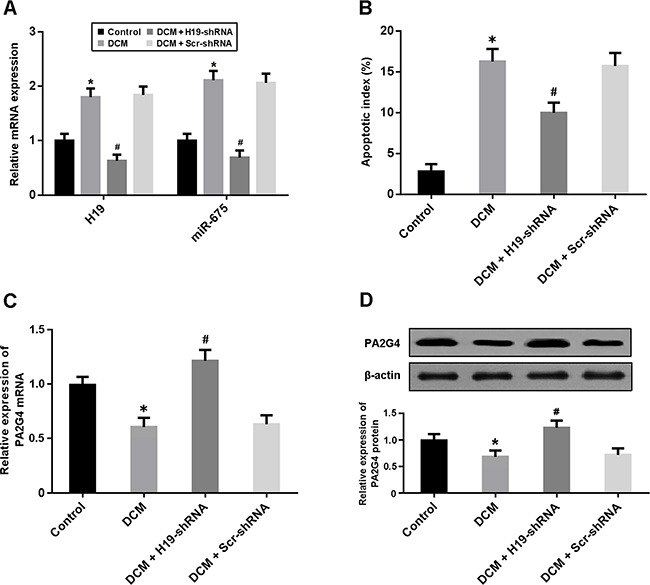
**(A)** The expression of H19 and miR-675 in myocardium was detected by real-time PCR. **(B)** Cardiomyocyte apoptosis was determined by TUNEL staining and the apoptotic index was calculated. **(C, D)** The mRNA and protein expression of PA2G4 was detected by real-time PCR and Western blot. *P < 0.05, vs. Control; ^#^ P < 0.05, vs. DCM (n = 5).

The expression of H19 and miR-675 was significantly upregulated in the myocardium of DCM rats and downregulated after administration of lentivirus H19-shRNA (Figure 1A). Cardiomyocyte apoptosis was evaluated by TUNEL staining and apoptotic index was increased in the DCM group and decreased in the DCM + H19-shRNA group (Figure 1B). Moreover, the mRNA and protein expression of PA2G4 was reduced in DCM rats and elevated following treatment with lentivirus H19-shRNA (Figure 1C, 1D).

**Figure 2:**
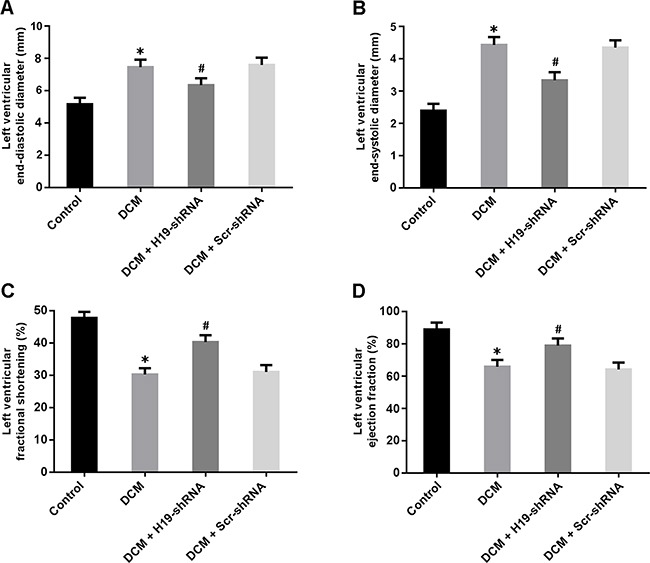
Cardiac structure and function were evaluated by echocardiography **(A)** Left ventricular end-diastolic diameter; **(B)** Left ventricular end-systolic diameter; **(C)** Left ventricular fractional shortening; **(D)** Left ventricular ejection fraction. * P < 0.05, vs. Control; ^#^ P < 0.05, vs. DCM (n = 5).

Cardiac structure and function were measured by echocardiography. LVEDD and LVESD were significantly increased in the DCM group and decreased in the DCM + H19-shRNA group (Figure 2A, 2B). In addition, LVFS and LVEF were found to be reduced in the DCM rats, whereas knockdown of H19 could markedly improve left ventricular systolic function (Figure 2C, 2D).

**Figure 3:**
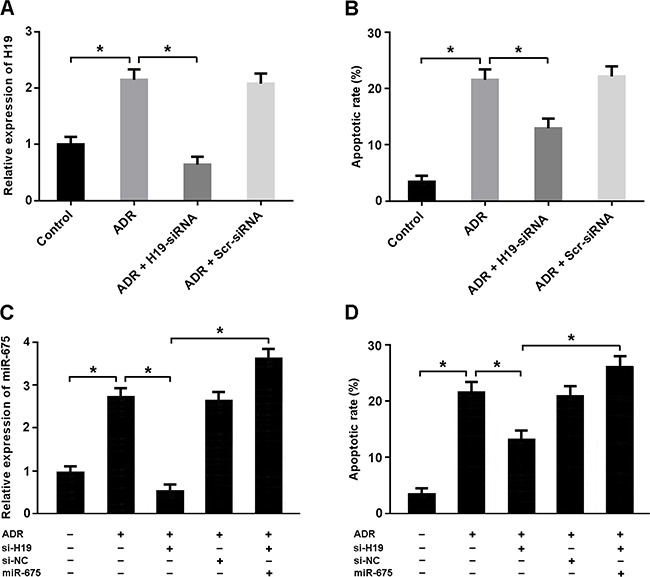
**(A, B)** Cardiomyocytes were transfected with adenoviral H19-siRNA or scramble-siRNA and then exposed to adriamycin (ADR, 1 μmol/L) for 24 h. The H19 expression was detected by real-time PCR and the apoptotic rate was determined by flow cytometry. **(C, D)** Cardiomyocytes were transfected with adenoviral H19-siRNA and/or miR-675 mimic and then treated with ADR (1 μmol/L) for 24 h. The miR-675 expression was analyzed by real-time PCR and the apoptotic rate was measured by flow cytometry. * P < 0.05 (n = 3 independent experiments).

Adriamycin treatment was associated with increased H19 expression and cardiomyocyte apoptosis, while H19-siRNA transfection was found to suppress apoptosis (Figure 3A, 3B). The expression of miR-675 was upregulated in cardiomyocytes treated with adriamycin. Enforced expression of miR-675 could increase apoptosis in cardiomyocytes with adriamycin treatment and H19-siRNA transfection (Figure 3C, 3D).

**Figure 4:**
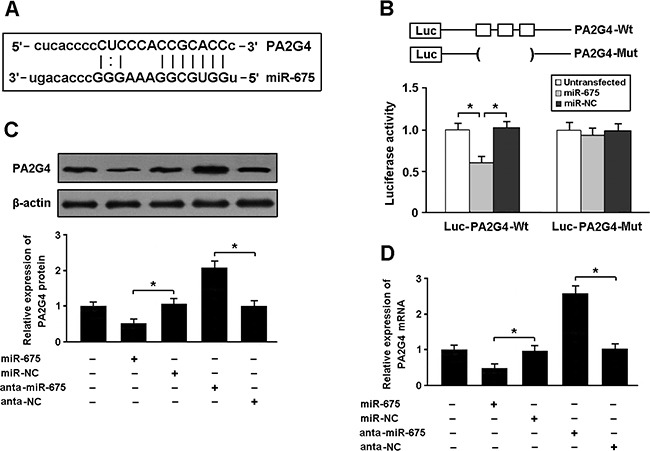
**(A)** PA2G4 was predicted as a target gene of miR-675 using miRBase. **(B)** HEK293 cells were transfected with miR-675 mimic and luciferase constructs of PA2G4 3′UTR or mutant, and luciferase activity assay was performed 48 h after transfection. **(C, D)** Cardiomyocytes were transfected with miR-675 mimic or antagomir, and the expression of PA2G4 was determined by real-time PCR and Western blot. * P < 0.05 (n = 3 independent experiments).

To determine whether PA2G4 is the target gene of miR-675, we conducted luciferase reporter assay in HEK293 cells. Our results showed that PA2G4 was a direct target of miR-675 (Figure 4A, 4B). The expression of PA2G4 was reduced in cardiomyocytes transfected with miR-675 mimic and increased after transfected with miR-675 antagomir (Figure 4C, 4D).

**Figure 5:**
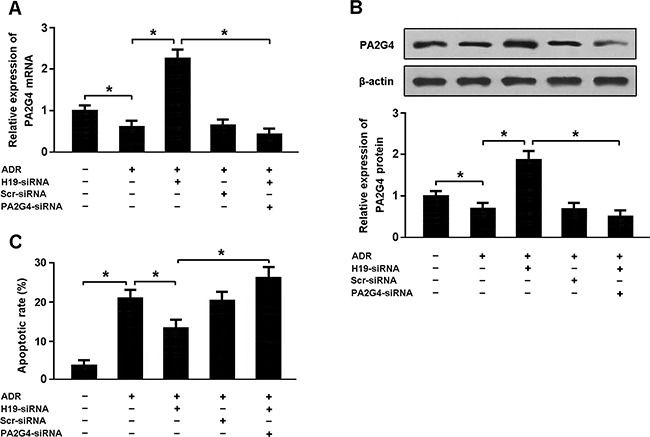
**(A, B)** Cardiomyocytes were transfected with H19-siRNA and/or PA2G4-siRNA followed by treatment with adriamycin (ADR, 1 μmol/L) for 24 h, and the expression of PA2G4 was analyzed using real-time PCR and Western blot. **(C)** Cardiomyocytes were stained with Annexin V/PI and then subjected to flow cytometry to determine apoptosis. * P < 0.05 (n = 3 independent experiments).

Cardiomyocytes were transfected with H19-siRNA and PA2G4-siRNA followed by treatment with adriamycin and cellular apoptosis was detected by flow cytometry (Figure 5A–5C). Our results indicated that H19 knockdown could increase PA2G4 expression and reduce apoptosis in cardiomyocytes exposed to adriamycin. Moreover, downregulation of both H19 and PA2G4 was found to promote apoptosis in cardiomyocytes treated with adriamycin.

## DISCUSSION

DCM is the most common cause of congestive heart failure in young patients. Recently, significant progress has been made in the medical treatment of DCM. However, the molecular mechanisms of DCM are still not fully understood. In this study, we established a rat model of adriamycin-induced DCM and found that the expression of lncRNA H19 was significantly upregulated. We then performed *in vivo* and *in vitro* experiments to explore the potential roles of H19 in the pathogenesis of DCM. Our results demonstrated that overexpression of H19 was associated with increased cardiomyocyte apoptosis in DCM rats. H19 was found to induce myocardial apoptosis by upregulating miR675, which consequently inhibited the expression of anti-apoptosis gene PA2G4.

H19 is a highly conserved imprinted transcript and is actively involved in the embryonic development and growth regulation [4]. The multiple functions of H19 are illustrated by its interaction with miRNAs. It has been reported that H19 can act as a competing endogenous RNA to sponge miR-106a and the miR-let7 family members [5, 6]. In addition, H19 can also function as a precursor of miR-675 to post-translationally modulate several target genes involved in various cell processes [7–9]. In this study, our findings indicated that H19 was upregulated in the myocardium of DCM rats and H19/miR-675 axis was associated with cardiomyocyte apoptosis.

miRNAs are a class of endogenous small non-coding RNAs that negatively regulate gene expression at post-transcriptional level by binding to the 3′ UTR of the target mRNA, leading to translational inhibition or mRNA degradation [10]. It has been well documented that miRNAs are critically involved in the regulation of a variety of biological processes, including cell differentiation, proliferation and apoptosis. In this study, we found that both miR-675 and its precursor H19 were upregulated in cardiomyocytes exposed to adriamycin. Moreover, the results of luciferase reporter assay suggested that PA2G4 might be a direct target of miR-675.

Cardiomyocyte apoptosis plays an important role in the pathogenesis of adriamycin-induced DCM [2]. PA2G4, also known as EBP1, is a potential regulator of ErbB3 signaling, and is implicated in cell growth, apoptosis and differentiation. EBP1 is highly conserved throughout evolution and is structurally homologous to the methionine aminopeptidases. In recent years, there is growing evidence that EBP1 can exert anti-apoptotic effects in various pathophysiological processes [11–13]. In the present study, our findings suggested that H19/miR-675 axis could participate in the regulation of adriamycin-induced cardiomyocyte apoptosis by targeting EBP1.

In conclusion, our study revealed a novel function of H19/miR-675/PA2G4 pathway in the regulation of cardiomyocyte apoptosis, which will provide valuable insights into understanding the pathological mechanisms of adriamycin-induced DCM.

## MATERIALS AND METHODS

### Animal model and grouping

All experiments were approved by the Animal Ethics Committee of Soochow University and were carried out in accordance with the Guide for the Care and Use of Laboratory Animals. The rat model of DCM was established by using adriamycin. Male Sprague-Dawley rats weighing 200-250g were housed in a room at 22 ± 2°C and 50 ± 5% relative humidity with an alternating 12-h light/dark cycle. Adriamycin was intraperitoneally administered to rats in six equal injections (each containing 2.5 mg/kg) over a period of 3 weeks for a total cumulative dose of 15 mg/kg. The rats were randomly divided into 4 groups: control group, DCM group, DCM + H19-shRNA group (DCM rats intracoronary injected with lentivirus H19-shRNA), and DCM + Scr-shRNA group (DCM rats injected with scramble shRNA). After 4 weeks, animals were sacrificed by cervical dislocation, and the hearts were harvested for analysis.

### Cardiomyocyte culture

Neonatal rat cardiomyocytes were isolated and cultured as previously described [14]. Briefly, the hearts were surgically removed from 1-2 days old rats and myocardial tissues were cut into small pieces and then underwent a series of digestion at 37°C in D-Hanks solution containing 1.2 mg/mL pancreatin and 0.14 mg/mL collagenase (GIBCO, USA). After centrifugation, the cells were suspended in Dulbecco's modified Eagle's medium (GIBCO, USA) containing 20% calf serum, 100 U/ml penicillin and 100 μg/ml streptomycin. The dissociated cells were preplated at 37°C for 1 h to separate cardiomyocytes by adherence of cardiac fibroblasts. Thereafter, cells were collected and diluted to 1×10^6^ cells/ml and plated onto gelatin-coated culture dishes.

### Echocardiographic study

Echocardiography was performed after 4 weeks of lentivirus administration as previously described [15]. Left ventricular end-diastolic diameter (LVEDD) and left ventricular end-systolic diameter (LVESD) were measured from the parasternal long-axis view. Left ventricular fractional shortening (LVFS) and left ventricular ejection fraction (LVEF) were detected to assess left ventricular systolic function.

### TUNEL staining

Cardiomyocyte apoptosis was determined by the terminal deoxynucleotidyl transferase-mediated dUTP nick-end labeling (TUNEL) assay. The apoptotic index was calculated as the percentage of TUNEL-positive cells divided by the total number of cells. TUNEL *in situ* cell death detection kits were purchased from Promega Biotec.

### Flow cytometry

Cardiomyocytes were stained with Annexin V-FITC and propidium iodide (BD Pharmingen, USA), and then subjected to flow cytometry to determine apoptosis. The apoptotic rate was calculated as the percentage of Annexin V-positive and propidium iodide-negative cells divided by the total number of cells in the gated region.

### Luciferase reporter assay

To determine whether PA2G4 was a direct target of miR-675, we performed luciferase reporter experiments in HEK293 cells. The 3′-untranslated region (UTR) of PA2G4 was cloned into the downstream of luciferase gene to generate Luc-PA2G4-Wt vector. The 3′-UTR without predicted miR-675 binding site was constructed to generate Luc-PA2G4-Mut vector. For luciferase assay, cells were transfected with either wild-type or mutant construct with and without miRNA mimic. Luciferase activity was determined 48 h after transfection using the Luciferase Reporter Assay System (Promega, USA).

### Real-time PCR

Total RNA was isolated from cardiac tissue using TRIzol reagent (Invitrogen, USA). The RNA was then reverse transcribed using SuperScript First-Strand cDNA System (Invitrogen, USA) according to the manufacturer's instructions. Real-time reactions were run and analyzed by using a Real-Time PCR system (Applied Biosystems 7500). The relative expression of mRNA was calculated using the comparative cycle threshold (CT) (2^−ΔΔCT^) method with GAPDH as the endogenous control to normalize the data.

### Western blotting

Proteins were prepared from myocardial tissues and neonatal cardiomyocytes. The protein extracts were subjected to centrifugation at 12 000 g for 15 min, loaded onto SDS-PAGE gels, and then transferred to nitrocellulose membranes. After blocking with 5% non-fat milk in Tris-buffered saline, the membranes were incubated with primary antibodies overnight at 4 °C, followed by incubation with HRP-conjugated secondary antibodies. The antibodies were purchased from Cell Signaling Technology and were used at manufacturer-recommended dilutions. Finally, the signal was detected using the enhanced chemiluminescence kit (Amersham Biosciences, USA).

### Statistical analysis

In this study, data were presented as mean ± SD. Statistical comparisons between multiple groups were performed using one-way ANOVA, followed by Dunnet's post hoc test. *P* < 0.05 was considered statistically significant.
